# Perinatal Outcomes Associated with Latency in Late Preterm Premature Rupture of Membranes

**DOI:** 10.3390/ijerph18020672

**Published:** 2021-01-14

**Authors:** Eui Kyung Choi, So Yeon Kim, Ji-Man Heo, Kyu Hee Park, Ho Yeon Kim, Byung Min Choi, Hai-Joong Kim

**Affiliations:** 1 Department of Pediatrics, Division of Neonatology, Korea University College of Medicine, Seoul 02841, Korea; ekchoi03@korea.ac.kr (E.K.C.); czrabbit@korea.ac.kr (K.H.P.); cdmin@korea.ac.kr (B.M.C.); 2 Department of Obstetrics and Gynecology, Korea University College of Medicine, Seoul 02841, Korea; specialsy@kumc.or.kr (S.Y.K.); heojiman.md@gmail.com (J.-M.H.); haijkim@korea.ac.kr (H.-J.K.)

**Keywords:** preterm premature rupture of membranes 1, late preterm 2, preterm birth 3, expectant management 4, antenatal corticosteroids 5, neonatal sepsis 6, respiratory distress syndrome 7

## Abstract

This study aims to evaluate the perinatal outcomes of preterm premature rupture of membrane (PPROM) with latency periods at 33 + 0–36 + 6 weeks of gestation. This retrospective case-control study included women with singleton pregnancies who delivered at 33 + 0–36 + 6 weeks at Korea University Ansan Hospital in South Korea between 2006–2019. The maternal and neonatal characteristics were compared between different latency periods (expectant delivery ≥72 h vs. immediate delivery <72 h). Data were compared among 345 women (expectant, *n* = 39; immediate delivery, *n* = 306). There was no significant difference in maternal and neonatal morbidities between the groups, despite the younger gestational age in the expectant delivery group. Stratified by gestational weeks, the 34-week infants showed a statistically significant lower exposure to antenatal steroids (73.4% vs. 20.0%, *p* < 0.001), while the incidence of respiratory distress syndrome (12.8%) and the use of any respiratory support (36.8%) was higher than those in the 33-week infants, without significance. Our study shows that a prolonged latency period (≥72 h) did not increase maternal and neonatal morbidities, and a considerable number of preterm infants immediately delivered at 34 weeks experienced respiratory complications. Expectant management and antenatal corticosteroids should be considered in late preterm infants with PPROM.

## 1. Introduction

Preterm premature rupture of membranes (PPROM), defined as the rupture of membranes before 37 weeks of gestation, accounts for one-third of all preterm births [1]. When PPROM occurs between 23 + 0 and 34 + 0 weeks of gestation, expectant management in women without evidence of infection is used as the standard form of care, with suggested delivery at 34 + 0 weeks of gestation if labor has not yet ensued. However, the optimal time for delivery beyond 34 + 0 weeks of gestation with PPROM is still considered controversial. 

The management of PPROM depends on the balance between the risk of ascending infection and those associated with prematurity. Previously, three trials (PROMEXIL, PROMEXIL 2, and PPROMT) comparing active and expectant management in women with PPROM at 34 + 0 and 36 + 6 weeks of gestation did not show that immediate delivery reduces the rate of early onset neonatal sepsis. Furthermore, late preterm (LPT) infants in the immediate delivery group were more often diagnosed with respiratory distress syndrome (RDS) [2,3,4]. Current emerging evidence suggests that LPT infants born at 34 + 0–36 + 6 weeks of gestation have increased morbidity or mortality, with possible long-term neurodevelopmental consequences secondary to their late prematurity, followed by economic burden worldwide [5,6,7]. 

Recently, a large, randomized, controlled trial reported that the administration of betamethasone significantly reduced the rate of neonatal respiratory complications in LPT infants [8]. Based on this, guidelines set out by the American College of Obstetricians and Gynecologists (ACOG) recommend the administration of betamethasone for pregnant women at risk of having a LPT birth between 34 + 0 and 36 + 6 weeks of gestation [9]. These infants may benefit from expectant management because of the advantage of lung maturation through the complete course of betamethasone administration. 

To address this issue, we analyzed the effect of latency on perinatal outcomes at 33 + 0–36 + 6 weeks of gestation, with PPROM based on latency of <72 h vs. ≥72 h. Furthermore, we investigated whether expectant management longer than 72 h improved outcomes in LPT infants with PPROM according to gestational age.

## 2. Materials and Methods 

This study was conducted at a single tertiary institution between January 2006 and December 2016. We retrospectively reviewed the medical records of women with singleton pregnancies who delivered at 33 + 0–36 + 6 weeks of gestation at Korea University Ansan Hospital in South Korea. All recorded clinical characteristics, laboratory results, treatments, medications, and other procedures for the mothers and their neonates were examined. The pregnancies with known fetal malformations, multiple pregnancies, stillbirths, placenta previa, deliveries within 2 h of rupture, and cases where a decision to deliver was made due to other maternal or fetal indications were excluded. This study was approved by the Institutional Research Ethics Committee of the Korea University Ansan Hospital (2020ASS0211). 

Premature rupture of membrane (PROM) is diagnosed when the fetal membranes rupture before the onset of labor, while PPROM is the rupture of membranes before 37 weeks of gestation. The diagnosis of PROM was based on the observation of persistent vaginal pooling with positive nitrazine (blue strip indicating positive) and Actim PROM testing on sterile speculum examination. The Actim PROM test was used for detecting insulin-like growth factor-binding protein 1 in the vaginal fluid, with two blue lines on the dipstick indicating positive results. Gestational age was determined by the last menstrual period and confirmed by the first trimester ultrasound. The latency period was defined as the time interval between PROM and the time of delivery. 

Our institution followed the standard practice for the admission and management of PPROM, with a daily measurement of the vital signs, examination of uterine tenderness, nonstress test (thrice daily), complete blood counts and C-reactive protein (twice weekly), vaginal culture (once weekly), and fetal ultrasound (every two weeks). All patients received antibiotics for seven days, with a combination of intravenous ampicillin and erythromycin, according to the ACOG guidelines [9]. A single course of steroids, and if required, tocolytic agents, such as atosiban, beta-agonist, or magnesium (Mg), were administered before 34 weeks of gestation. Vaginal examination was conducted only if the patient was symptomatic or complained of contractions. The mode of delivery was decided based on the obstetric indications. 

Maternal characteristics including age, parity, gestational age at PPROM and delivery, and social factors (drinking alcohol and smoking) and their adverse pregnancy outcomes, such as incompetent internal os of the cervix, hypertensive disorder (preeclampsia, eclampsia, and chronic hypertension), diabetes mellitus (pregestational and gestational), oligohydramnios (amniotic fluid index ≤ 5 cm), and clinical and histologic chorioamnionitis were examined. Clinical chorioamnionitis was defined as the presence of uterine tenderness and/or foul-smelling amniotic fluid, maternal fever, leukocytosis, or fetal tachycardia with no other source of infection. Histologic chorioamnionitis was defined as the presence of acute chorioamnionitis, deciduitis, or funisitis according to the placental pathology report (histopathological evidence of the presence of acute inflammatory changes in the membrane roll and placental chorionic plate). 

Neonatal characteristics including gestational age, birth weight, small for gestational age (SGA; birth weight  <  10th percentile for age according to Fenton growth charts), and neonatal outcomes such as Apgar scores at 1 and 5 min, the need for resuscitation in the delivery room, treatment with exogenous surfactant, and ventilatory support (presence, duration, and type; invasive or noninvasive) were examined. The comorbidities of preterm infants, such as RDS (the presence of respiratory distress, increased oxygen requirement, and associated radiological findings), severe respiratory complications (a composite outcome of continuous positive airway pressure or high-flow nasal cannula for a minimum of 24 continuous hours, supplemental oxygen with a fraction of inspired oxygen of at least 0.30 for minimum of 24 continuous hours, extracorporeal membrane oxygenation or mechanical ventilation, stillbirth, or neonatal death within 72 h after delivery), patent ductus arteriosus and its treatment, intraventricular hemorrhage (grading according to Papile’s classification), early sepsis (either proven by bacterial culture or clinically highly suspected sepsis during the first 72 h of life), retinopathy of prematurity, and necrotizing enterocolitis (according to modified Bell’s criteria) were also assessed. Bronchopulmonary dysplasia was defined as the requirement of supplemental oxygen with a fraction of inspired oxygen of more than 0.21 for the first 28 days of life. Hypoglycemia was defined as a glucose level of less than 40 mg/dL (2.2 mmol/L) at any time.

A data analysis was performed using SPSS 20.0 for Windows (SPSS Inc., Chicago, IL, USA). The Shapiro–Wilk method was used to assess the normality of the data. Continuous variables were analyzed using either the *t*-test or the Mann–Whitney U-test for normal or skewed distributions. Proportions were tested using the chi-squared test and Fisher’s exact test. *p*-values of <0.05 were considered statistically significant. Data are presented as mean  ±  standard deviation (SD), median and interquartile range (IQR), or rate. The correlation between neonatal morbidity and gestational age was analyzed using linear-by-linear association by the chi-square test. Power calculations were performed to establish the sample size required to obtain statistical significance at the 95% level with 80% power (Power Analysis in R). All *p*-values were two-sided, and a *p*-value of less than 0.05 was considered significant.

## 3. Results

**Figure 1:**
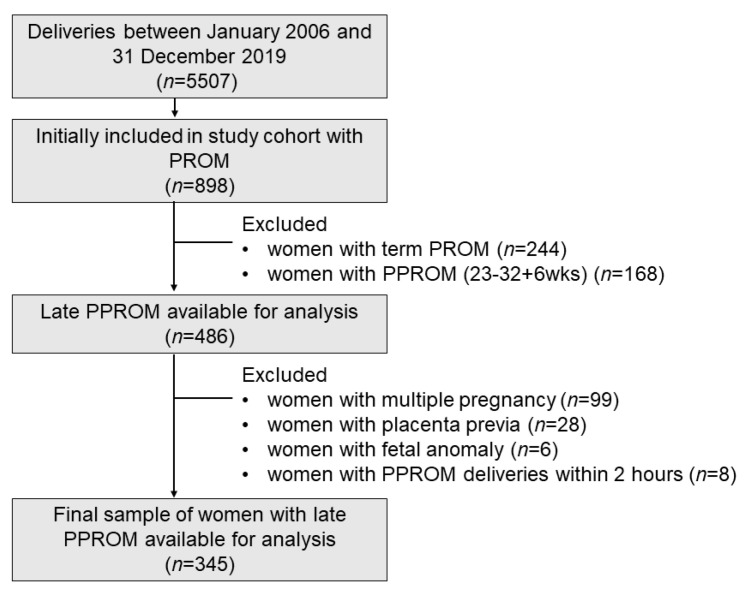
Flow chart of the study population.

Of the 5507 women who underwent deliveries between January 2006 and December 2016, 244 women with term PROM and 898 with PPROM were identified. Of the 486 women with preterm births between 33 + 0 and 36 + 6 weeks of gestation, 99 were excluded because of multiple pregnancies, 28 because of placenta previa, 8 because of deliveries within 2 h after PPROM, and 6 because of major fetal anomalies. The remaining 345 women and their infants were analyzed (Figure 1).

The demographic and clinical data of the women according to the latency period (72 h) are shown in Table 1. Among 345 women, 39 (11.3%) were managed expectantly (latency period ≥72 h), while 306 (88.7%) delivered immediately (latency period <72 h) after PPROM. The median gestational age at PPROM was shorter in the expectant group (33 + 2 weeks vs. 34 + 5 weeks, *p* < 0.001). Women with immediate deliveries had a greater history of abortion (20.5% vs. 45.5%, *p* = 0.003) and a shorter cervix (43.5% vs. 62.3%, *p* = 0.035). Women managed expectantly were treated with antenatal steroids (64.1% vs. 18.6%, *p* < 0.001), tocolytics (38.5% vs. 8.8%, *p* < 0.001), and Mg (17.9% vs. 3.3%, *p* = 0.001) and were more likely to undergo a cesarean delivery (59.0% vs. 40.3%, *p* = 0.027). Clinical chorioamnionitis was seen in 7 women (17.9%) in the expectant group versus 13 (4.3%) in the immediate delivery group (*p* = 0.004), but there was no significant difference between the two groups for histological chorioamnionitis (*p* = 0.361).

**Figure 2:**
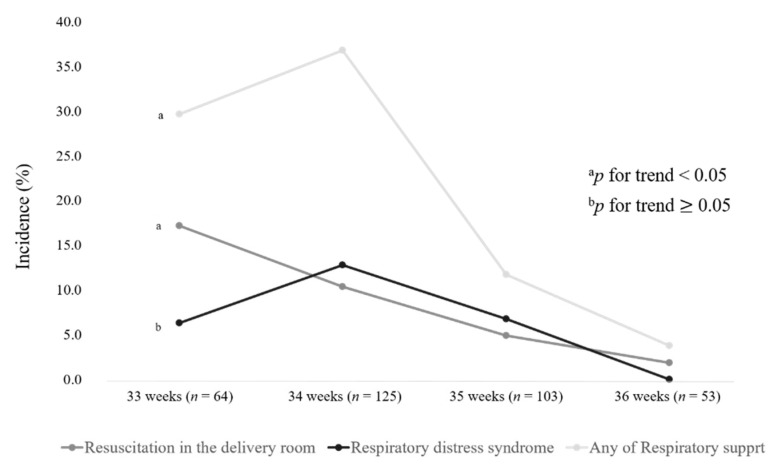
Linear-by-linear association of neonatal outcomes according to gestational age.

Table 2 shows the neonatal outcomes according to the latency period (72 h). There were significant differences in the median gestational age (34 + 1 weeks vs. 34 + 6 weeks, *p* < 0.001), but there were no significant differences between the two groups, including early-onset sepsis and neonatal morbidities, for other neonatal outcome measures. As shown in Table 3 and Table 4, a stratified analysis was performed to detect any differences in the effect of expectant management by gestational age (33, 34, 35, and 36 weeks of gestation). Antenatal steroids were administered to 73.4%, 20%, 4.9%, and 9.4% of women who delivered at 33, 34, 35, and 36 weeks of gestation, respectively. Compared with infants born at 33 weeks of gestation, infants born at 34 weeks of gestation showed a significantly lower exposure to antenatal steroids (73.4% vs. 20.0%, *p* < 0.001), while the incidence of RDS (6.3% vs. 12.8%, *p* = 0.166) and the use of any respiratory support (29.7% vs. 36.8%, *p* = 0.330) and conventional ventilator (1.6%, vs. 5.6%, *p* = 0.270) were higher without significance. However, the LPT infants born at 35 and 36 weeks of gestation required significantly less respiratory assistance and had less severe respiratory complications than those born at 33 and 34 weeks of gestation. Figure 2 shows the linear-by-linear association of neonatal outcomes according to the gestational age. The linear-by-linear association test indicated no significant decrease in the incidence of RDS with the increase in gestational age (*p* for trend = 0.082). 

When neonatal outcomes were assessed according to latency by gestational age, there was a significant difference in the frequency of antenatal steroid administration according to the latency period in the 34-week gestation group (78.6% vs. 12.6%, *p* < 0.001) (Table 4). RDS was less frequently diagnosed in the expectant group than in the immediate group at 34 weeks (7.1% vs. 13.5%, *p* = 0.693) and 35 weeks of gestation (0.0% vs. 7.3%, *p* = 1.000) without statistical significance, with the less marked difference at 33 and 36 weeks of gestation. Early sepsis was 3.1% (2/64), 1.6% (2/125), 1% (1/103), and 0% (0/53) at 33 weeks, 34 weeks, 35 weeks, and 36 weeks of gestation, respectively, with no significant differences according to the latency. This section may be divided by subheadings. It is intended to provide a concise and precise description of the experimental results, their interpretation, as well as the experimental conclusions that can be drawn.

## 4. Discussion

In the present study, we found that at 33 + 0–36 + 6 weeks of gestation after PPROM, the adverse perinatal outcomes of mothers with a long latent period (>72 h) did not increase, except for clinical chorioamnionitis. Furthermore, a long latent period resulted in comparable adverse neonatal outcomes, including respiratory morbidity and sepsis, compared with the short latent period (≤72 h), despite the younger gestational age. Stratified by gestational weeks, a considerable number of newborns born at 34 weeks of gestation had RDS and required assisted respiration when compared with those born at 33 weeks of gestation, but the results were not significant. In addition, the frequency of antenatal steroid administration was significantly higher, and RDS was less frequently diagnosed, in the expectant group at 34 weeks.

Our results were consistent with a recent metanalysis concluding that expectant management improved maternal and infant outcomes in late preterm PROM, specifically relating to maternal infection [10]. Previous studies have noted a strong association between chorioamnionitis and longer latency [11]. Our results also noted that longer latency was associated with significantly higher rates of clinical chorioamnionitis. Nevertheless, there were no reported adverse events related to clinical chorioamnionitis, increased histologic chorioamnionitis, length of hospital stays, or systemic infection, while all patients with PPROM were given the same antibiotics. This administration of prophylactic antibiotic and the immediate delivery when a sign of infection developed might be related to no further complications during and after delivery, although the rate of clinical chorioamnionitis was higher with longer latency. However, the balance between the beneficial effect of latency prolongation and the adverse outcomes of chorioamnionitis should be further assessed. 

A number of studies have been done on the varying latencies after PPROM and the impact of latency on perinatal outcomes [2,3,12,13]. A recent metanalysis demonstrated no contraindications to expectant management before 37 weeks of gestation with careful monitoring [10]. However, these studies did not stratify gestational weeks and did not address the significant respiratory issues present at 34 weeks of gestation without the assistance of steroids, as our work pointed out. We have, for the first time, demonstrated that differences in perinatal outcomes at each week of gestation after PPROM occur in the late preterm period. 

One of the major outcomes related to neonatal morbidity in LPT infants is respiratory problems, and we demonstrated RDS rates of 6.3%, 12.8%, 6.8%, and 0.0% at 33, 34, 35, and 36 weeks of gestation, respectively. Recently, there have been a few studies on the differences in respiratory morbidities among LPT infants at different gestational ages, although their data were limited by the relatively small sample size [14,15,16]. In the present study, RDS in the infants born at 34 weeks of gestation was comparable to the incidence of 13.5% reported by Robertson et al. [17]. Although this notable finding was not statistically significant, the incidence in the infants after PPROM born at 34 weeks of gestation with RDS and requiring respiratory assistance was greater than those born at 33 weeks. This was probably because the prenatal steroid administration and expectant management were emphasized in less than 34 weeks of gestation. This result closely aligned with those of earlier studies that reported late preterm infants not equivalent to their term counterparts, especially those born between 34 + 0 and 34 + 6 weeks of gestation [15]. 

Previous trials demonstrated an improvement in neonatal respiratory outcomes with betamethasone, followed by a significant reduction in RDS in late preterm infants exposed to antenatal steroids [18] and a decrease in health care costs [19]. However, these studies included all LPT births, regardless of various causes of preterm birth or PPROM. A regression analysis of another study showed that the incidence of RDS in PPROM prior to 37 weeks of gestation decreased with antenatal steroid use [20], but this study did not stratify each gestational week in LPT birth. 

Our study is the first report on LPT infants with PPROM to detect any differences in the effect of expectant management and to compare neonatal outcomes at 33 and 34 weeks of gestation stratified by antenatal steroid. The major strength of this study is the clinically relevant population of complicated LPT infants with PROM, and our results represent a better obstetrical practice. 

However, this study has several limitations. First, a small number of subjects with a latency of >72 h were enrolled because the standard practice of PPROM after 34 weeks of gestation has been immediate delivery. The lack of any significant difference may have been attributed to our small sample size. The required sample size of each group to show a significant difference in respiratory distress rate was *n* = 2390. Further correlations to RDS rates may be limited by insufficient power in this study. Secondly, this is a retrospective approach with possible biases on the part of the perinatal specialists regarding different decisions on delivery timing after 34 weeks of gestation. Thirdly, our results are not generalizable, as this study was comprised of Asian subjects, and the hospital settings in this study might be different from those of others. 

## 5. Conclusions

We conclude that expectant management beyond 34 weeks of gestation with PPROM and the use of antenatal steroids during the late preterm period should be considered when there is no evidence of infection. Sufficient counseling should focus on the risks and benefits for mothers and babies with the immediate or expectant management of late preterm PROM. A large, prospective trial is needed, and guidelines should be developed for patients and practitioners. 

## Figures and Tables

**Table 1:** Maternal characteristics and pregnancy outcomes ^a^.

	Expectant Management > 72 h (*n* = 39)	Immediate Delivery ≤ 72 h (*n* = 306)	*p* Value
Gestational age at PPROM (weeks)	33 ^+ 2^ (32 ^+ 4^–33 ^+ 6^)	34 ^+ 5^ (34 ^+ 1^–35 ^+ 3^)	0.000
Latency period (h)	130 (84–218)	15 (7–32)	0.000
Maternal age (y)	32.5 (29–35)	31 (28–34)	0.260
Alcohol (%)	0 (0.0)	2 (0.7)	1.000
Smoking (%)	0 (0.0)	10 (3.3)	0.611
Nulliparity (%)	20 (51.3)	185 (60.5)	0.272
Preterm history (%)	4 (10.3)	21 (6.9)	0.506
Abortion history (%)	8 (20.5)	137 (45.4)	0.003
Cervical length < 25 mm (%)	17 (43.6)	175 (62.3)	0.035
IIOC (%)	1 (2.6)	12 (3.9)	1.000
Preeclampsia (%)	2 (5.1)	27 (8.9)	0.758
Diabetes mellitus (%)	3 (7.7)	38 (12.5)	0.598
Oligohydramnios (%)	8 (20.5)	69 (23.4)	0.840
Antenatal antibiotics (%)	39 (100)	295 (96.7)	0.611
Antenatal steroid (%)	25 (64.1)	57 (18.6)	0.000
Tocolytics (%)	15 (38.5)	27 (8.8)	0.000
Mg (%)	7 (17.9)	10 (3.3)	0.001
Induction (%)	16 (42.1)	105 (35.4)	0.474
Cesarean delivery (%)	23 (59.0)	123 (40.3)	0.027
Placenta abruptio (%)	1 (2.6)	10 (3.3)	1.000
HCAM (%)	10 (47.6)	32 (36.8)	0.361
Funisitis (%)	1 (4.8)	5 (5.9)	1.000
Clinical CAM (%)	7 (17.9)	13 (4.3)	0.004

Abbreviations: PPROM, preterm premature rupture of membranes; IIOC, incompetent internal os of cervix; Mg, magnesium; HCAM, histologic chorioamnionitis; CAM, chorioamnionitis. ^a^ Data are presented as median and interquartile range (IQR) or rate.

**Table 2:** Neonatal outcomes stratified by expectant management ^a^.

	Expectant Management > 72 h (*n* = 39)	Immediate Delivery ≤ 72 h (*n* = 306)	*p* Value
Gestational age	34 ^+ 1^ (33 ^+ 3^–34 ^+ 6^)	34 ^+ 6^ (34 ^+ 1^–35 ^+ 4^)	0.000
Birth weight (weeks)	2,468 (2,230–2,705)	2,429 (2,219–2,638)	0.504
Small for gestational age (%)	1 (2.6)	16 (5.2)	0.705
Male (%)	21 (53.8)	169 (55.2)	0.870
Apgar score at 5 min < 7 point (%)	1 (2.6)	9 (2.9)	1.000
Resuscitation in the delivery room (%)	4 (10.3)	26 (8.5)	0.761
Respiratory distress syndrome (%)	2 (5.1)	25 (8.2)	0.753
Any respiratory support (%)	10 (25.6)	69 (22.5)	0.665
Severe respiratory complication (%)	6 (15.4)	45 (14.7)	0.910
Early-onset sepsis (%)	0 (0.0)	5 (1.6)	1.000
Hypoglycemia (%)	7 (17.9)	53 (17.4)	0.929
Intraventricular hemorrhage (%)	0 (0.0)	2 (0.7)	1.000
Necrotizing enterocolitis (%)	1 (2.6)	0 (0.0)	0.113
Periventricular leukomalacia (%)	0 (0.0)	1 (0.3)	1.000
Bronchopulmonary dysplasia (%)	0 (0.0)	1 (0.3)	1.000
Retinopathy of prematurity (Grade >1) (%)			
Death (%)			
Hospital days (days)	12 (8–15)	11 (8–14)	0.079

^a^ Data are presented as median and interquartile range (IQR) or rate.

**Table 3:** Neonatal outcomes stratified by delivery week.

	33 Weeks (*n* = 64)	34 Weeks (*n* = 125)	35 Weeks (*n* = 103)	36 Weeks (*n* = 53)
Antenatal steroid (%)	47 (73.4)	25 (20.0) ^a^	5 (4.9) ^a^	5 (9.4) ^a^
Apgar score at 5 min < 7 point (%)	2 (3.1)	7 (5.6)	1 (1.0)	0 (0.0)
Resuscitation in the delivery room (%)	11 (17.2)	13 (10.4)	5 (4.9) ^a^	1 (1.9) ^a^
Respiratory distress syndrome (%)	4 (6.2)	16 (12.8)	7 (6.8)	0 (0.0)
Any respiratory support (%)	19(29.7)	46 (36.8)	12 (11.7) ^ab^	2 (3.8) ^ab^
Severe respiratory complication (%)	13 (20.3)	28 (22.4)	9 (8.7) ^ab^	1 (1.9) ^ab^
Early sepsis (%)	2 (3.1)	2 (1.6)	1 (1.0)	0 (0.0)

^a^ Compared with 33 weeks, *p* value < 0.05.; ^b^ Compared with 34 weeks, *p* value < 0.05.

**Table 4:** Neonatal outcomes in the immediate delivery versus expectant management groups stratified by delivery week.

	33 Weeks (*n* = 64)			34 Weeks (*n* = 125)			35 Weeks (*n* = 103)			36 Weeks (*n* = 53)	
	Expectant (*n* = 15)	Immediate (*n* = 49)	*p* Value	Expectant (*n* = 14)	Immediate (*n* = 111)	*p* Value	Expectant (*n* = 7)	Immediate (*n* = 96)	*p* Value	Expectant (*n* = 3)	Immediate (*n* = 50)
Steroid (%)	12 (80.0)	35 (71.4)	0.740	11 (78.6)	14 (12.6)	0.000	1 (14.3)	4 (4.2)	0.302	1 (33.3)	4 (8.0)
Apgar (%)	1 (6.7)	1 (2.0)	0.417	0 (0.0)	7 (6.3)	1.000	0 (0.0)	1 (0.9)	1.000		
Resusci (%)	2 (13.3)	9 (18.4)	1.000	1 (7.1)	12 (10.8)	1.000	1 (14.3)	4 (4.2)	0.302	0 (0)	1 (2.0)
RDS (%)	1 (6.7)	3 (6.1)	1.000	1 (7.1)	15 (13.5)	0.693	0 (0)	7 (7.3)	1.000		
R support (%)	14 (28.6)	5 (33.3)	0.753	5 (35.7)	41 (36.9)	1.000	0 (0.0)	12 (12.5)	1.000		2 (4.0)
Severe C (%)	10 (20.4)	3 (20.0)	1.000	3 (21.4)	25 (22.5)	1.000	0 (0.0)	9 (9.4)	1.000		1 (2.0)
Hypog (%)	3 (20.0)	15 (30.6)	0.525	3 (21.4)	18 (16.2)	0.704	1 (14.3)	14 (14.6)	1.000	0 (0)	6 (12.0)
Early S (%)	2 (4.1)	0 (0.0)	1.000	0 (0.0)	1 (0.9)	1.000	0 (0.0)	1 (0.9)	1.000		

Apgar, Apgar score at 5 min < 7 points; Resusci, Resuscitation; RDS, Respiratory distress syndrome; R, Respiratory; C, Complication; Hypog, Hypoglycemia; S, Sepsis.

## Data Availability

The data presented in this study are available on request from the corresponding author.
